# Theranostics in Hematooncology

**DOI:** 10.2967/jnumed.122.265199

**Published:** 2023-07

**Authors:** Andreas K. Buck, Sebastian E. Serfling, Sabrina Kraus, Samuel Samnick, Niklas Dreher, Takahiro Higuchi, Leo Rasche, Hermann Einsele, Rudolf A. Werner

**Affiliations:** 1 Department of Nuclear Medicine, University Hospital Würzburg, Würzburg, Germany;; 2 Department of Internal Medicine II, University Hospital Würzburg, Würzburg, Germany; and; 3 Russell H. Morgan Department of Radiology and Radiological Science, Johns Hopkins University School of Medicine, Baltimore, Maryland

**Keywords:** theranostics, C-X-C motif chemokine receptor 4, CXCR4, lymphoma, radioimmunotherapy, hematooncology

## Abstract

In the early 2000s, major clinical trials provided evidence of a favorable outcome from antibody-mediated radioimmunotherapy for hematologic neoplasms, which then led to Food and Drug Administration approval. For instance, the theranostic armamentarium for the referring hematooncologist now includes ^90^Y-ibritumomab tiuxetan for refractory low-grade follicular lymphoma or transformed B-cell non-Hodgkin lymphoma, as well as ^131^I-tositumomab for rituximab-refractory follicular lymphoma. Moreover, the first interim results of the SIERRA phase III trial reported beneficial effects from the use of ^131^I-anti-CD45 antibodies (Iomab-B) in refractory or relapsed acute myeloid leukemia. During the last decade, the concept of theranostics in hematooncology has been further expanded by C-X-C motif chemokine receptor 4–directed molecular imaging. Beyond improved detection rates of putative sites of disease, C-X-C motif chemokine receptor 4–directed PET/CT also selects candidates for radioligand therapy using β-emitting radioisotopes targeting the identical chemokine receptor on the lymphoma cell surface. Such image-piloted therapeutic strategies provided robust antilymphoma efficacy, along with desired eradication of the bone marrow niche, such as in patients with T- or B-cell lymphoma. As an integral part of the treatment plan, such radioligand therapy–mediated myeloablation also allows one to line up patients for stem cell transplantation, which leads to successful engraftment during the further treatment course. In this continuing education article, we provide an overview of the current advent of theranostics in hematooncology and highlight emerging clinical applications.

Fueled by the favorable results of prospective clinical trials, recent years have witnessed a more widespread adoption of prostate-specific membrane antigen–targeted theranostics (1*,*^2^) or somatostatin receptor–targeted theranostics (3*,*^4^). These molecular image–guided therapeutic approaches have focused on solid tumor entities, such as prostate carcinoma or neuroendocrine neoplasms (1*,*^4^), but there is also a growing body of evidence of favorable outcomes in hematooncology (5–7). For instance, with roots back to the 80s (8), radioimmunotherapy exploits the concept of monoclonal antibodies labeled with radioisotopes, thereby allowing for β-emission mediated by antigenic binding sites that are overexpressed on the tumor cell surface but not in unaffected tissue (9*,*^10^). In this regard, radiolabeled CD20 antibodies that are conjugated to ^90^Y or ^131^I have been extensively evaluated in clinical trials (5*,*^6^), leading to overall response rates of up to 80% in patients with B-cell lymphoma (6) and durable remissions for years (11). Not surprisingly, these beneficial results of radioimmunotherapy then led to the Food and Drug Administration approval of nonmyeloablative antibody-mediated hot treatments, including ^90^Y-ibritumomab tiuxetan (Zevalin; Acrotech Biopharma) for refractory low-grade follicular lymphoma or transformed B-cell non-Hodgkin lymphoma (NHL), as well as ^131^I-tositumomab (Bexxar; GlaxoSmithKline) for rituximab-refractory follicular lymphoma (12*,*^13^). In patients scheduled for radioimmunotherapy, pretherapeutic imaging has also allowed estimation of absorbed doses to tumor and normal organs, thereby rendering radioimmunotherapy a true theranostic approach (6*,*^14^).

In the last decade, however, novel peptide-based tracers targeting the C-X-C motif chemokine receptor 4 (CXCR4) have been applied in varying hematooncologic scenarios, including ^68^Ga-pentixafor for imaging and ^177^Lu-/^90^Y-pentixather for treatment (7*,*^15^*,*^16^). In a physiologic context, CXCR4 may emerge as a promising theranostic target. First, it is crucially involved in homing of stem and progenitor cells and in hematopoiesis (17*,*^18^). Second, in a pathophysiologic context, this G-protein–coupled receptor and its ligand stromal cell–derived factor 1 also mediate metastatic spread via various subcellular mechanisms, including paracrine stimulation of angiogenesis or migration of CXCR4-positive tumor cells to other organs with increasing stromal cell–derived factor 1 expression (17). As such, CXCR4-seeking radiotracers for imaging and therapy can leverage these physiologic and pathophysiologic aspects to improve diagnostic accuracy or determine the chemokine receptor extent before CXCR4-directed radioligand therapy (RLT). Systemic whole-body irradiation can then bring about antilymphoma cell kill and bone marrow (BM) eradication for hematopoietic stem cell transplantation (HSCT), in particular when combined with established radioimmunotherapeutics (NHL; Fig. 1) (7*,*^19^).

**FIGURE 1.**
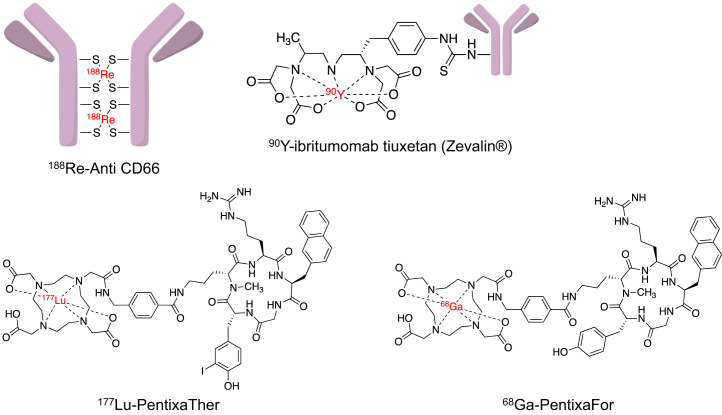
Structures of established (^188^Re-CD66 antibodies, ^90^Y-ibritumomab tiuxetan) and novel (^68^Ga-pentixafor, ^90^Y-pentixather) theranostic agents applied in hematooncology.

In the present review, we provide an overview of extensively tested radiolabeled immunotherapies and introduce the growing clinical applications of novel CXCR4-mediated theranostics in hematooncology.

## RADIOIMMUNOTHERAPY

### Concept and Targets

In patients with lymphoma, varying targets on disease manifestations have been exploited on a cellular level to deliver β-emitting radiation. For B-cell lymphoma, these include designated antigens, in particular CD20, CD22, and CD37 (9). In this article, we focus on major clinical trials that triggered Food and Drug Administration approval for selected compounds, including ^90^Y-ibritumomab tiuxetan and ^131^I-tositumomab. We also highlight recent favorable results for ^131^I-anti-CD45 antibodies (^131^I-anti-CD45-apamistamab [Iomab-B]; Actinium Pharmaceuticals), which are currently being tested in a phase III trial on acute myeloid leukemia (AML) (20).

### NHL

**FIGURE 2.**
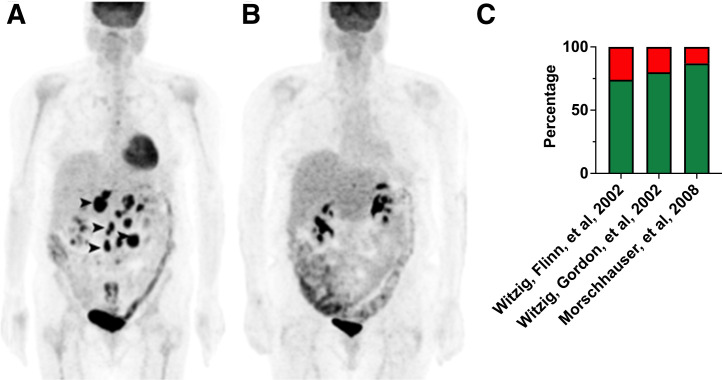
Patient with NHL treated with ^90^Y-ibritumomab tiuxetan. (A) Pretherapeutic maximum-intensity projection derived from ^18^F-FDG PET revealed multiple lymphoma manifestations in abdomen (arrowheads). (B) PR with inactive disease was achieved as visualized on ^18^F-FDG PET at 3-mo follow-up. (Modified from (67).) (C) Response rates of major clinical trials using ^90^Y-ibritumomab tiuxetan (6*,*^22^*,*^25^) (green indicates disease control depending on study’s definition; red indicates uncontrolled disease).

DeNardo et al. were among the first to apply fractionated radioimmunotherapy to refractory NHL that had been subjected to a mean of 4 previous treatment lines. ^131^I-labeled Lym-1, a monoclonal antibody interacting with class II histocompatibility antigens, led to a complete response (CR) in 33%, with a mean duration of 1.2 y, along with activity-dependent myeloablation (21). Mainly spearheaded by Witzig et al., clinical trials on rituximab-refractory NHL led to the approval of CD20-targeting ^90^Y-ibritumomab tiuxetan. Pretreatment with rituximab ensured B-cell depletion; radioimmunotherapy followed, which led to CR in 15% and a partial response (PR) in 59% (overall response rate, 74%) (22). Enrolling subjects with relapsed, refractory, or transformed CD20-positive NHL, the same research group reported on a phase III trial comparing ^90^Y-ibritumomab tiuxetan with rituximab serving as a cold reference. Objective response rates were significantly higher for radioimmunotherapy (80%) than for rituximab (56%), with CR in 30% of the patients scheduled for ^90^Y-ibritumomab tiuxetan (vs. only 16% in the rituximab arm). The radioimmunotherapy off-target effect most often recorded was BM toxicity with reversible myelosuppression (6). Figure 2A shows a patient with NHL achieving PR after injection of ^90^Y-ibritumomab tiuxetan, along with the response rates in selected clinical trials evaluating radioimmunotherapy in lymphoma patients (Fig. 2B).

Kaminski et al. were among the first to evaluate the antilymphoma efficacy of the ^131^I-labeled murine anti-CD20 monoclonal antibody tositumomab in patients with refractory or transformed NHL. When compared with a patient’s last qualifying chemotherapy, a single injection of the hot compound led to disease control (PR or CR) in 65%, whereas the last chemotherapy achieved such a favorable outcome in only 28%, indicating that a single ^131^I-tositumomab treatment is highly effective (23). In subjects experiencing progressive disease under rituximab, a phase II trial then demonstrated overall and CR rates of 65% and 38%, respectively. Median progression-free survival was more than 2 y in responders to radioimmunotherapy (24).

### Follicular Lymphoma

In a phase III trial, patients with advanced stage III or IV follicular lymphoma in the first remission were randomized into a radioimmunotherapy arm (consisting of rituximab over 7 d, followed by ^90^Y-ibritumomab tiuxetan) or no treatment. CD20-targeted radioimmunotherapy doubled progression-free survival, with a high PR-to-CR rate, leading to a final response rate of 87%. Again, the most commonly observed side effects were hematologic, with a grade of at least 3 in 8% (25). In a follow-up study evaluating long-term response, the time to the next treatment was 8.1 y for patients who had received radioimmunotherapy, a time that was significantly prolonged when compared with the control arm without treatment (time to next treatment, 3 y) (26). In a phase III trial initiated by the Southwest Oncology Group and by Cancer and Leukemia Group B (SWOG S0016), 554 treatment-naïve subjects with advanced follicular lymphoma received cyclophosphamide, doxorubicin, vincristine, and prednisone (CHOP) along with immunotherapy using cold rituximab (CHOP-R). In the comparative arm, CHOP was combined with ^131^I-tositumomab consolidation (CHOP-radioimmunotherapy). Over 24 mo, however, both protocols achieved comparable progression-free survival (CHOP-R, 76%, vs. CHOP-radioimmunotherapy, 80%) and overall survival rates (CHOP-R, 97%, vs. CHOP-radioimmunotherapy, 93%) (27).

### Diffuse Large B-Cell Lymphoma

Recent efforts also turned toward the use of ^90^Y-ibritumomab tiuxetan in diffuse large B-cell lymphoma patients for whom HSCT has failed—a clinical scenario associated with poor prognosis (28). As such, Lugtenburg et al. exploited synergistic effects using ^90^Y-ibritumomab tiuxetan along with rituximab, prednisolone, etoposide, chlorambucil, and lomustine. Such combination treatments achieved 1-y survival in almost half of these difficult-to-treat patients (28).

### Acute Myeloid Leukemia (AML)

Using a combination regimen of Iomab-B, fludarabine, and 2 Gy of total-body irradiation, Pagel et al. reported on 58 patients (with either AML or high-risk myelodysplastic syndrome) in a phase II trial demonstrating complete remission in all subjects, followed by successful HSCT (29). The currently recruiting phase III SIERRA trial will then shed light on the beneficial use of Iomab-B in relapsed and refractory AML by comparing this agent with conventional care. Because of an increasing rate of comorbidities, HSCT in the elderly AML patient is conducted with caution (30), and in the SIERRA trial, this issue will be addressed. Relapsed or refractory AML patients at least 55 y old receive either conventional care or Iomab-B, and subjects treated with conventional care can cross over to radioimmunotherapy. An interim analysis reported on 63 patients allocated to the conventional-care arm, and of those, 11 (17.4%) achieved CR and were then scheduled for HSCT. The remaining 52 subjects (83%) did not achieve a response; thus, 38 crossed over to Iomab-B. All patients with Iomab-B then received HSCT, which led to engraftment. This was independent of whether they had initially been randomized into the Iomab-B arm (60/60; 100%) or whether they crossed over (38/38; 100%). The rate of sepsis was also lower in the radioimmunotherapy group than in subjects with conventional-care–mediated HSCT. As such, this interim report indicated that Iomab-B conditioning enabled HSCT even in subjects for whom approved conventional care failed, led to successful neutrophile reconstitution, and was associated with fewer side effects than conventional-care–based transplantation (31).

### Reasons for Declining Use of Radioimmunotherapy

Both ^90^Y-ibritumomab tiuxetan and ^131^I-tositumomab are associated with extensive costs—approximately $25,000 for a single injection—leading to reimbursement challenges in the United States and Europe. Despite the remarkable outcome benefits, this obstacle may partially explain the declining application of radioimmunotherapy using ^90^Y-ibritumomab tiuxetan or ^131^I-tositumomab in recent years (32). Radioimmunotherapy can also cause long-term adverse effects on BM function, including a severe decrease in platelets and leukocytes or the occurrence of myelodysplastic syndrome in selected cases (33). Moreover, in recent years, novel and effective therapies have also entered the clinical arena, such as CAR T-cell therapies or bispecific T-cell engagers (34).

## PEPTIDE-MEDIATED THERANOSTICS

### Concept and Targets

Mediating angiogenesis and tumor cell dissemination along with resistance to treatment, chemokine receptors have emerged as an attractive pan-hematologic cancer target (17*,*^35^). For instance, in marginal zone lymphoma (MZL), AML, B-cell chronic lymphocytic leukemia, or multiple myeloma (MM), CXCR4 expression may have prognostic value (36–39). Targeting this chemokine receptor in patients with hematologic neoplasms may offer a better rate of detection of putative sites of disease or even determine a high risk of therapeutic or chemotherapeutic resistance.

**FIGURE 3.**
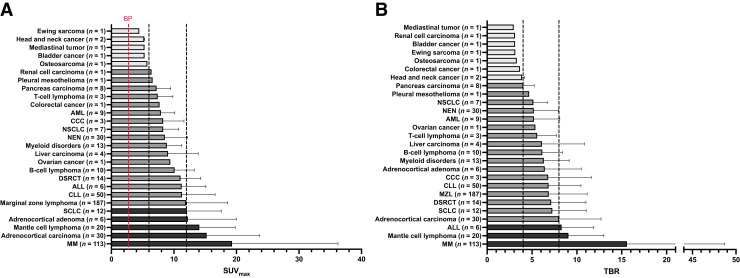
SUV_max_ (A) and target-to-background ratios (B) for 690 patients scanned with ^68^Ga-pentixafor PET/CT for assessment of in vivo CXCR4 extent. Black dashed lines show SUV_max_ of 6 and 12 and target-to-background ratio of 4 and 8, respectively. ALL = acute lymphoblastoid leukemia; BP = blood pool; CCC = cholangiocarcinoma; CLL = chronic lymphocytic leukemia; DSRCT = desmoplastic small round cell tumor; NEN = neuroendocrine neoplasm; NSCLC = non–small cell lung carcinoma; SCLC = small cell lung carcinoma; adrenocortical adenoma = aldosterone-producing adrenocortical adenoma. (Modified from (40).)

First, to evaluate the diagnostic performance, a recent study pooled retrospective data on the PET agent ^68^Ga-pentixafor in 690 subjects scheduled for 777 scans. Among all tested tumor entities (in total, *n* = 35), hematologic malignancies revealed the highest in vivo CXCR4 expression (determined by SUV_max_) and elevated target-to-background ratios. For solid cancers, however, only small cell lung and adrenocortical carcinomas showed an increased SUV_max_ and target-to-background ratio (Fig. 3) (40). As such, ^68^Ga-pentixafor may emerge as a pan-hematologic tumor agent, in particular for MM, MZL, and leukemia.

On the basis of these favorable imaging results, patients were also scheduled for chemokine receptor–directed RLT. In this regard, administration of pentixather causes myeloablation due to CXCR4-moderated maintenance of hematopoietic stem progenitor cells in the BM (41). Such a pentixather-mediated myeloablation, however, can be used to prepare the patient for HSCT as an integral component of the treatment algorithm. Pretherapeutic dosimetry using ^177^Lu-pentixather allowed for a scintigraphically visible accumulation of radiotracer in normal organs. Absorbed doses to the hepatic or splenic parenchyma were acceptable, with a range of 0.6–0.7 Gy/GBq, whereas for the kidneys, as the dose-limiting organ, the reported dose was 0.9 Gy/GBq of ^177^Lu-pentixather, corresponding to 3.8 Gy/GBq of ^90^Y-pentixather (42). The commonly applied limit of 23 Gy for renal tissue is therefore not exceeded (43), which would be reached after 20–30 GBq of ^177^Lu-pentixather (5–8 GBq of ^90^Y-pentixather) (42). These doses, however, could be reduced through coinfusion of nephroprotective amino acids (44), and chemokine receptor–directed RLT is also normally restricted to 1 cycle. On-target doses in lymphoma tissue are substantial (42) and, thus, may also be associated with other relevant off-target effects due to lymphoma cell kill. For instance, Maurer et al. reported side effects among a broad range of patients with hematologic malignancies who were scheduled for last-line CXCR4-directed RLT in a salvage setting. Right after treatment, vital signs were normal, indicative of no acute toxicity. Further corroborating previous reports, however, a substantial fraction of patients died from neutropenic sepsis or progressive disease before successful engraftment after HSCT (45). To avoid these lethal events, countermeasures have been incorporated, including protocols to prevent tumor lysis syndrome before initiation of RLT (19). Another elegant approach exploits the physical properties of the used radionuclides. The short half-life of ^90^Y (2.7 d) led to significantly reduced intervals between CXCR4 RLT and the onset of conditioning regimens, particularly when compared with the β-emitting alternative ^177^Lu (6.7 d). The aplastic phase was thus reduced, thereby avoiding life-threatening infections (45).

### MM

**FIGURE 4.**
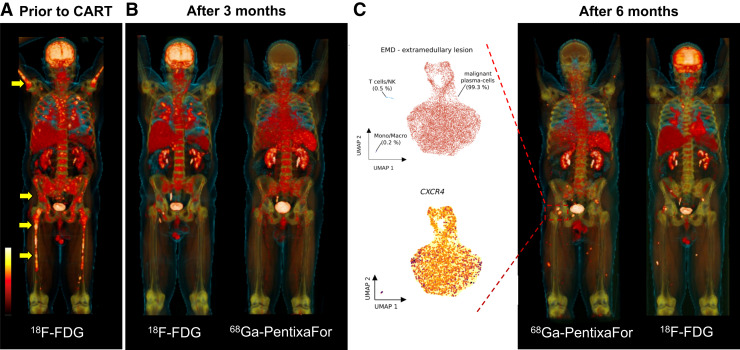
CXCR4-targeting ^68^Ga-pentixafor PET/CT for dissecting true relapse and autoimmune-mediated side effects in MM patient scheduled for B-cell maturation antigen–targeting CAR T-cell therapy (idecabtagene vicleucel). (A) Before CAR T-cell therapy, ^18^F-FDG showed osseous lesions (arrows). (B) On restaging 3 mo after CAR T-cell therapy, myeloma clearance in skeleton was observed on maximum-intensity projections of ^18^F-FDG and ^68^Ga-pentixafor PET. Only ^18^F-FDG, however, revealed uptake in pulmonary system; no such radiotracer accumulation was observed on CXCR4-directed imaging. Single-cell RNA sequencing on lung specimen demonstrated upregulation of Th17.1-positive T cells, which are associated with autoimmune diseases such as sarcoidosis. (C) Six months after CAR T-cell therapy, both imaging modalities showed novel manifestations (red box) suggestive of relapse. ^68^Ga-pentixafor PET–guided biopsy was conducted, and single-cell RNA sequencing then revealed malignant plasma cells along with increased CXCR4 expression (leftmost panel in C). CART = CAR T-cell therapy. (Modified from (15).)

In MM, CXCR4 triggers the onset of distant manifestations, such as by osteoclastogenesis and multidrug resistance (46), suggesting that targeting of this receptor may provide not only an improved diagnostic read-out but also prognostic capabilities (39). When subjects who were scheduled for a lesion-based comparison of ^18^F-FDG and ^68^Ga-pentixafor were investigated, the latter agent detected more MM manifestations in 21% (the 2 agents were equal in 42%, and ^18^F-FDG was superior in the remaining 37%). CXCR4-targeted PET positivity was also associated with survival, with negative findings on PET being linked to improved outcome. This was even more pronounced for subjects showing no extramedullary lesions on ^68^Ga-pentixafor PET/CT. A substantially elevated SUV_max_ has been recorded, indicating that CXCR4-targeted RLT is feasible in MM (47). Providing further evidence of the role of ^68^Ga-pentixafor as a noninvasive biomarker of disease activity, a recent prospective trial reported associations between uptake in disease sites with end-organ damage and the extent of β2-microglobulin, serum free light chains, and urine light chains (48). We also investigated the usefulness of CXCR4-directed molecular imaging in the context of pseudoprogression under CAR T-cell therapy as a strategy to disentangle immune-mediated causes for such flare-ups from true progression. Relative to ^18^F-FDG, chemokine receptor PET was able to differentiate between an autoimmune phenomenon and a true relapse, with single-cell RNA sequencing of biopsy samples serving as a reference. First, 3 mo after CAR T-cell therapy, ^68^Ga-pentixafor PET in the lung was negative. Respective biopsies then revealed Th17.1 T-helper cells associated with a sarcoidotic reaction, suggestive of pseudoprogression. Six months after treatment, however, ^68^Ga-pentixafor PET was then positive in novel extramedullary lesions, which also showed high CXCR4 expression on single-cell RNA sequencing, indicative of a true relapse (Fig. 4) (49).

Given the intense radiotracer accumulation after administration of ^68^Ga-pentixafor in MM, the theranostic counterpart ^177^Lu-pentixather was first applied in 3 subjects with heavily pretreated, advanced MM with intra- and extramedullary manifestations. In 2 of these individuals, a short-term response with reduced uptake on follow-up ^18^F-FDG PET/CT was recorded, indicative of therapeutic benefit (50). Another 8 MM patients were then scheduled for CXCR4-directed RLT, and myeloma doses of up to 70 Gy were reported, with CR in 1 patient and PR in 5 subjects (overall survival, 7.5 mo). Another patient, however, died of sepsis during the aplastic phase, whereas the remaining individual experienced lethal tumor lysis caused by RLT (51).

### MZL

**FIGURE 5.**
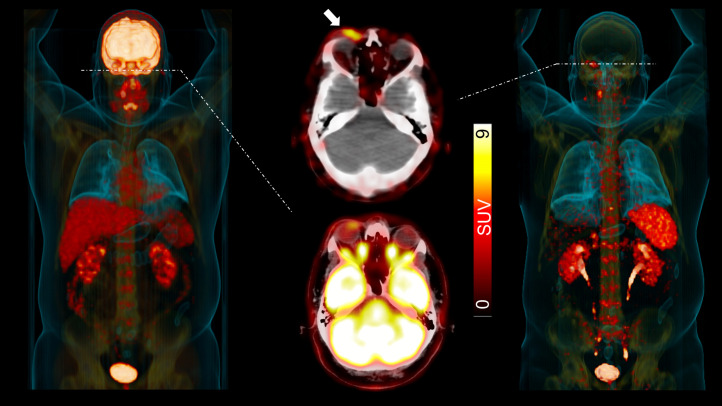
MZL patient with additional periorbital disease site (white arrow) identified on ^68^Ga-pentixafor PET/CT. On ^18^F-FDG maximum-intensity projection (MIP, left) and transaxial PET/CT (middle, bottom), periorbital manifestation was masked by normal biodistribution in brain. On CXCR4-targeted ^68^Ga-pentixafor (MIP, right; transaxial PET/CT, middle top), this additional site of disease can be identified because of missing brain accumulation. (Modified from (53).)

**FIGURE 6.**
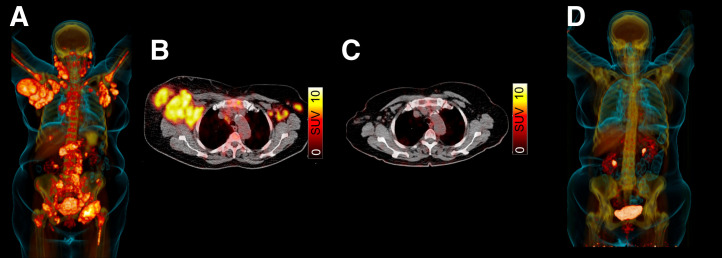
Patient with MZL and scheduled for rituximab-bendamustine. (A and B) Maximum-intensity projection (A) of ^68^Ga-pentixafor PET revealed multiple lymphoma manifestations, in particular in axilla as seen on transaxial PET/CT (B). (C and D) After treatment, complete remission was achieved on follow-up imaging, indicating that CXCR4-targeted PET/CT may be useful to monitor treatment response. (Modified from (53).)

In a recent ex vivo analysis investigating extranodal MZL (or mucosa-associated lymphoid tissue [MALT] lymphomas), chemokine receptor expression was recorded in virtually all cases, whereas somatostatin receptors (as another theranostic target) were absent in half the samples (52). Duell et al. were among the first to evaluate the diagnostic benefit for imaging of MZL and investigated varying subtypes, including 22 patients with extranodal, nodal, and splenic origin. When compared with guideline-compatible routine diagnostic procedures (colonoscopy, BM biopsy, and CT as part of hybrid imaging using ^18^F-FDG PET), ^68^Ga-pentixafor detected all true-positive and all true-negative cases (22/22) whereas conventional staging was correct in only 17 of the investigated subjects (Fig. 5). The latter radiotracer identified advanced disease (Ann Arbor stage ≥ 3) in more than half the patients, which led to an upstaging in 7 of 22 (31.8%) and a change in treatment in 8 of 22 (36.4%). These modifications in oncologic management included intensified treatment in 6 of 8 (75%) (53). Future studies should also evaluate the role of assessing treatment response (53), such as under chemotherapeutic regimens (Fig. 6). These retrospective investigations triggered further prospective phase I/II trials focusing on MALT lymphomas. Mayerhoefer et al. enrolled 26 patients with a gastric disease origin and determined the value of CXCR4 PET/CT for assessing incomplete remission on follow-up after guideline-compatible eradication of *Helicobacter pylori* (54*,*^55^). ^68^Ga-pentixafor PET and MRI were conducted on all subjects, and comparison with biopsy-derived specimens revealed accuracy of 97%, specificity of 100%, and a slightly lower sensitivity of 95% (54*,*^55^). Thus, to identify residual disease during follow-up, ^68^Ga-pentixafor PET may replace the currently recommended intense diagnostic work-up of invasive procedures, including endoscopy and histologic assessments twice per year (54*,*^55^). To date, however, CXCR4-directed RLT has not been applied to MZL.

### Leukemia and Lymphoma

Patients with AML may benefit from CXCR4-directed molecular imaging because of the origin of this disease in the protective BM niche, along with the antileukemia effects of CXCR4 antagonists (56*,*^57^). Herhaus et al. first investigated the primary blasts of patients and reported on an association of blast counts with CXCR4 upregulation using flow cytometry. In a dedicated animal model, ^68^Ga-pentixafor small-animal PET was positive only in CXCR-positive, not CXCR4-negative, xenografts, whereas in patients with AML, PET positivity was noted in half the subjects, which was further corroborated on MRI (58). PET positivity in AML, however, may be exploited to identify candidates for disrupting CXCR4/CXCL12 interactions, such as plerixafor as an adjunct to chemotherapeutic regimens (59*,*^60^). In a prospective setup, ^68^Ga-pentixafor PET/MRI was also used in chronic lymphocytic leukemia (61), as CXCR4 has been advocated to play a crucial role in BM infiltration in this leukemia subtype (62). When compared with solid tumors or other types of hematologic malignancies (MALT), the highest SUVs were recorded in the BM in this patient population, indicating that ^68^Ga-pentixafor may be useful for biopsy planning (61).

**FIGURE 7.**
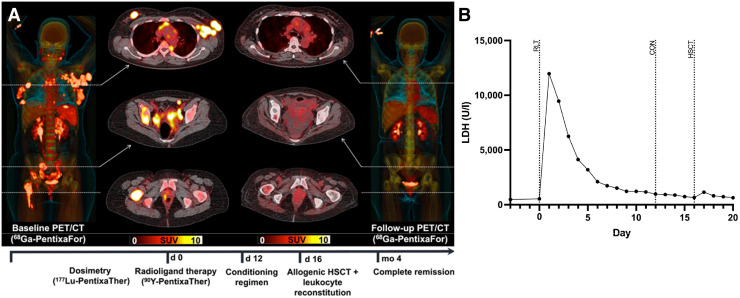
Patient with relapsed T-cell lymphoma treated with CXCR4-directed RLT and achieving complete remission. (A) Maximum-intensity projection and transaxial ^68^Ga-pentixafor PET/CT at baseline showed extensive disease in skeleton and lymph nodes. Four months after treatment, CR was noted on follow-up ^68^Ga-pentixafor PET/CT. (B) Lactate dehydrogenase as surrogate marker of antilymphoma efficacy peaked directly after RLT and then rapidly declined till conditioning regimen and HSCT, thereby suggesting direct antilymphoma effect caused by CXCR4 RLT. CON = conditioning regimen; LDH = lactate dehydrogenase. (Modified from (7).)

**FIGURE 8.**
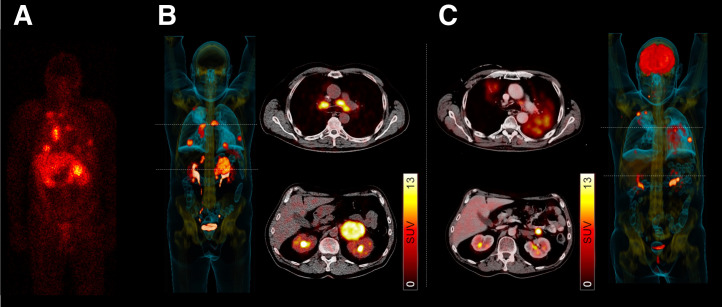
Synergistic effects of radioimmunotherapy and CXCR4-targeted RLT in patient with heavily pretreated diffuse large B-cell lymphoma. (A) Pretherapeutic scintigraphy 24 h after ^177^Lu-pentixafor injection revealed multiple disease sites, allowing for calculations of absorbed doses. (B) Baseline maximum-intensity projection and transaxial ^68^Ga-pentixafor PET/CT showed multiple CXCR4-expressing mediastinal and abdominal lesions. (C) ^90^Y-ibritumomab tiuxetan combined with ^90^Y-pentixather was initiated. On ^18^F-FDG PET/CT 4 mo later, sites of disease were smaller, indicating PR. (Modified from (63).)

CXCR4-directed RLT was then also applied to AML and patients with lymphoma. In patients with relapsed T-cell lymphoma, doses in extramedullary lesions ranged from 17.4 to 33.2 Gy, exerting relevant antilymphoma efficacy as revealed by longitudinal monitoring of lactate dehydrogenase. All 4 treated patients were also scheduled for chemotherapeutic conditioning or high-dose therapy. Lactate dehydrogenase had already peaked shortly after injection of ^177^Lu-pentixather (but before the onset of additional conditioning), suggesting a direct antilymphoma effect mediated by CXCR4 RLT. One of 4 patients died of septicemia 16 d after RLT, whereas the remaining 3 achieved disease control (PR or CR) with successful leukocyte reconstitution during follow-up. Patients with a favorable outcome were also scheduled for additional radioimmunotherapy using ^188^Re-labeled anti-CD66 (Fig. 7) (7). Also investigating a small case series of 6 patients with relapsed diffuse large B-cell lymphoma, Lapa et al. reported that 2 died of central nervous system aspergillosis and sepsis. In the remaining subjects, PR was again noted in those individuals who also received concomitant radioimmunotherapy. RLT-mediated eradication of the BM niche then also led to full engraftment after HSCT (63). The respective pretherapeutic dosimetry and baseline ^68^Ga-pentixafor PET results for a patient with diffuse large B-cell lymphoma treated with such a tandem therapy (^90^Y-pentixather and ^90^Y-ibritumomab tiuxetan) is provided in Figure 8. PR was then observed 4 mo after treatment.

Last, in acute leukemia, an observational study reported on 3 subjects also treated with pentixather. Only in the patient who also received additional CD66-targeted radioimmunotherapy was long-lasting CR after RLT achieved (64).

### Future Directions

During the annual conference of the German Society for Hematology and Oncology in 2022, an expert panel of hematooncologists and nuclear medicine physicians discussed potential clinical applications of CXCR4-targeted theranostics. There were several key findings. First, CXCR4-targeted PET/CT may have the potential to emerge as a novel diagnostic reference standard in patients with MZL, including its use for disease monitoring, such as for identifying individuals prone to transformation to large B-cell lymphoma. Second, aggressive lymphomas with involvement of the central nervous system may benefit from CXCR4-directed PET/CT, as the use of ^18^F-FDG is hampered by the physiologic biodistribution of ^18^F-FDG in the central nervous system (65). Third, CXCR4-targeted RLT may be most promising in patients with T-cell lymphoma, as case series reported favorable outcomes in these otherwise difficult-to-treat patients (7*,*^66^). Finally, the expert panel concluded that prospective studies on imaging of MZL and treatment of T-cell lymphoma are urgently needed.

Taken together, increasing levels of evidence on chemokine receptor–targeted imaging and therapy will guide toward implementation in national and international guidelines, ultimately leading to more widespread clinical use of CXCR4-directed theranostics. Titration studies should be conducted as a first step to determine the most appropriate activity for RLT (for both antilymphoma and myeloablative effects or for lymphoma cell kill only). These should be followed by multicenter phase II trials on the safety and efficacy of CXCR4 RLT alone. Last, competitive or additive concepts should be tested, such as through sequential tandem treatment approaches using chemokine receptor RLT and CAR T-cell therapies (15).

## CONCLUSION

Given the favorable results in major clinical trials, antibody-mediated radioimmunotherapy has been approved by the Food and Drug Administration for patients with refractory follicular lymphoma or transformed B-cell NHL. Inadequate reimbursement in Europe and the United States, however, has restricted more widespread adoption in clinical routine. The interim results of the SIERRA phase III trial, however, showed a favorable outcome from using Iomab-B in relapsed or refractory AML and may soon trigger a revival of radioimmunotherapy. CXCR4-targeted molecular imaging has been extensively evaluated across different hematologic and solid neoplasms, and the results indicate that ^68^Ga-pentixafor may emerge as a novel pan-hematologic tumor agent. For CXCR4-targeted PET/CT, promising applications include MM and MZL, whereas refractory T-cell lymphoma may benefit from CXCR4 RLT. Patients treated with chemokine receptor–targeting radiotherapeutics also experience the desired myeloablation, which then allows scheduling for HSCT. Such an eradication of the BM niche is then an integral component of the therapeutic algorithm beyond antilymphoma effects in selected cases. Last, observational studies also hinted that synergism may be achieved when combining CD20- or CD66-directed radioimmunotherapy with CXCR4-targeted RLT in advanced disease.
